# Collagen-derived dipeptide Pro-Hyp administration accelerates muscle regenerative healing accompanied by less scarring after wounding on the abdominal wall in mice

**DOI:** 10.1038/s41598-021-98407-9

**Published:** 2021-09-21

**Authors:** Shiro Jimi, Seiko Koizumi, Kenji Sato, Motoyasu Miyazaki, Arman Saparov

**Affiliations:** 1 Central Lab for Pathology and Morphology, Faculty of Medicine, Fukuoka University, 7-45-1 Nanakuma, Jonanku, Fukuoka, 8140180 Japan; 2 Nitta Gelatin Inc. R&D Center, Osaka, Japan; 3 Division of Applied Biosciences, Graduate School of Agriculture, Kyoto University, Kyoto, 606 8502 Japan; 4 Department of Pharmacy, Fukuoka University Chikushi Hospital, Fukuoka, Japan; 5 Department of Medicine, School of Medicine, Nazarbayev University, Nur-Sultan, Kazakhstan

**Keywords:** Biomaterials - proteins, Disease genetics, Muscle stem cells, Nutrition

## Abstract

Collagens act as cellular scaffolds in extracellular matrixes, and their breakdown products may also have important biological functions. We hypothesize that collagen dipeptide Pro-Hyp induces favorable healing activities and examined the effects of Pro-Hyp administered via different routes on wound healing using our novel murine model, in which an advanced fibrosis-prone scar lesion was developed in the abdominal muscle wall under the skin. After excising a part of the abdominal wall, a free-drinking experiment was performed using solutions with casein (CS), high molecular weight collagen peptides (HP), and low molecular weight collagen peptides including Pro-Hyp and Hyp-Gly (LP), in addition to water (HO). On day 21 of the study, when compared to the HO and CS groups, muscle regeneration in the LP group was significantly advanced in the granulation tissue, which was associated with a decrease in fibrosis. To clarify the effects of Pro-Hyp, daily intraperitoneal administration of pure Pro-Hyp was performed. Pro-Hyp administration induced many myogenically differentiated cells, including myogenin-positive myoblasts and myoglobin-positive myocytes, to migrate in the granulation tissue, while scar tissue decreased. These results indicated that Pro-Hyp administration accelerates muscle regenerative healing accompanied by less scarring after wounding on the abdominal wall.

## Introduction

Injury generates different responses in different organs. However, when tissue is extensively damaged by ulcers, injuries, and infarctions, granulation tissue is newly generated regardless of organ types, which is accompanied by robust inflammatory cell infiltration, fibroblast proliferation, neovascularization, and accumulation of extracellular matrix (ECM) including collagens^1,2^. Meanwhile, the wound is contracted by increased α-smooth muscle actin (αSMA) possessing myofibroblasts that appear and replicate in the granulation tissue by the effect of transforming growth factor-β (TGF-β)^3^. At the end phase, most of the cells in the tissue undergo apoptosis, and ECMs are degraded by matrix metalloproteases^1^, by which the granulation tissue is finally disappeared, and wound healing becomes terminated. However, sometimes, scar tissue remains after healing as a fibrous lesion, which consists of an excess deposition of collagens and matrix components^1,2^. The genesis of the fibrous lesions is supposed to be chronic inflammation, an overproduction of collagens and ECMs, and/or decreased degradation ability for the matrix proteins. Elevated scar lesions in the skin are classified as the matured scar, hypertrophic scar, and keloid, respectively^4,5^. These scar lesions usually occur in the skin tissue under a tensile force, namely mechanical stress^2,4,6^. Moreover, scar lesions occurred not only in the skin but also in other organs^7,8^, resulting in functional and morphological deterioration, causing a decrease in the quality of life of the patients.

Tissue regeneration is also important in wound healing^9^, and the granulation tissue is used as a scaffold for the regenerating cells. Human tissue regeneration is impacted by the quantity of granulation tissue, quality of ECMs, and the presence of regeneratable cells, and thus regenerative stem cell’s surroundings are important milieus for optimal healing. Strokes, spinal cord injuries, and myocardial infarctions are well recognized diseases containing cells that are unable to regenerate, such as central nerve cells, and cardiac muscle cells^10^. Though myocardial infarction is a persistent disease due to myocardial necrosis, other muscles such as smooth muscle and skeletal muscle can replicate and regenerate to some extents. In the case of skeletal muscle injury, satellite cells in the muscular fasciae are stimulated to replicate and differentiate into myoblasts, and they fuse and form myotubes, and thereafter they are functionally maturated into regenerated myofiber rich myocytes. These sequential morphological alterations in the myogenesis of the skeletal muscle have been intensively investigated^11^, but their responses in wound healing are still uncertain.

Out of all protein constituents in our body, collagen is ubiquitously distributes, and its amount is up to about 30%, and, in the skin, it reaches as high as about 70%^12^. Collagens are so far classified into 28 types^13^. The major types are: type I, which is rich in the dermis, ligament, and tendon, type II, which is found in the cartilage, type IV distributes in the basal membrane, and types I and III are found in the granulation tissue. Their structure and roles are different depending on location. Collagen-specific amino-acid residues are 3- and 4-hydroxy-proline (Hyp) and 5-hydroxy-lysine, which differ from other protein constituents^14^. In our previous wound healing study in mice, Pro-Hyp dipeptide was specifically generated during granulation tissue development after wounding^15^. It has been shown that collagen-derived peptides have biological activities^16^, and ingested hydrolysates accelerate wound healing reactions in patients with pressure ulcers^17^ and also enhance anti-aging improvements on the facial skin, including moisture, elasticity, wrinkles, and roughness^18^. Moreover, it has been shown that Pro-Hyp promotes cellular differentiation in osteoblastic cells^19^ and tendon cells^20^ by modulating intracellular signal pathways.

Scar lesions are hardly developed in rodents unless devices and chemicals are used^21–23^. However, we have established the wound healing model by excising a part of the abdominal wall in mice without any device or chemical, in which scar lesion and muscle regeneration simultaneously occur in the granulation tissue. In the present study, using the animal model, we performed two sets of animal studies to examine the effects of Pro-Hyp administered for 21 days by different methods, i.e., drinking and intraperitoneal (i.p.) injection. We focused on the morphological alterations of fibrous scar lesions and muscle differentiation in the granulation tissue in mice treated with Pro-Hyp and found that Pro-Hyp is useful in reducing scarring and increasing muscle regeneration in the granulation tissue on the abdominal muscle wall.

## Results

### Peptides drinking experiment

#### Bodyweight and hematological alterations

The experiment with different drinking solutions, i.e., water (HO) group, casein (CS) group, high molecular weight (5000–7000 Da) collagen peptides (HP) group, and low molecular weight (< 1000 Da) collagen peptides including Pro-Hyp and Hyp-Gly (LP) group, was performed immediately after the abdominal wall excision surgery. During the 21 days of the study, the solutions were freely available. Their consumption increased over time (Fig. S1), which may be due to their growth phase at a young age, and the CS group showed the highest consumption (about two-fold increase). However, no differences during the study were found in body weight, WBC, RBC, and platelet in the blood among the groups (Table S1).

#### Plasma level of collagen-derived peptides

**Figure 1:**
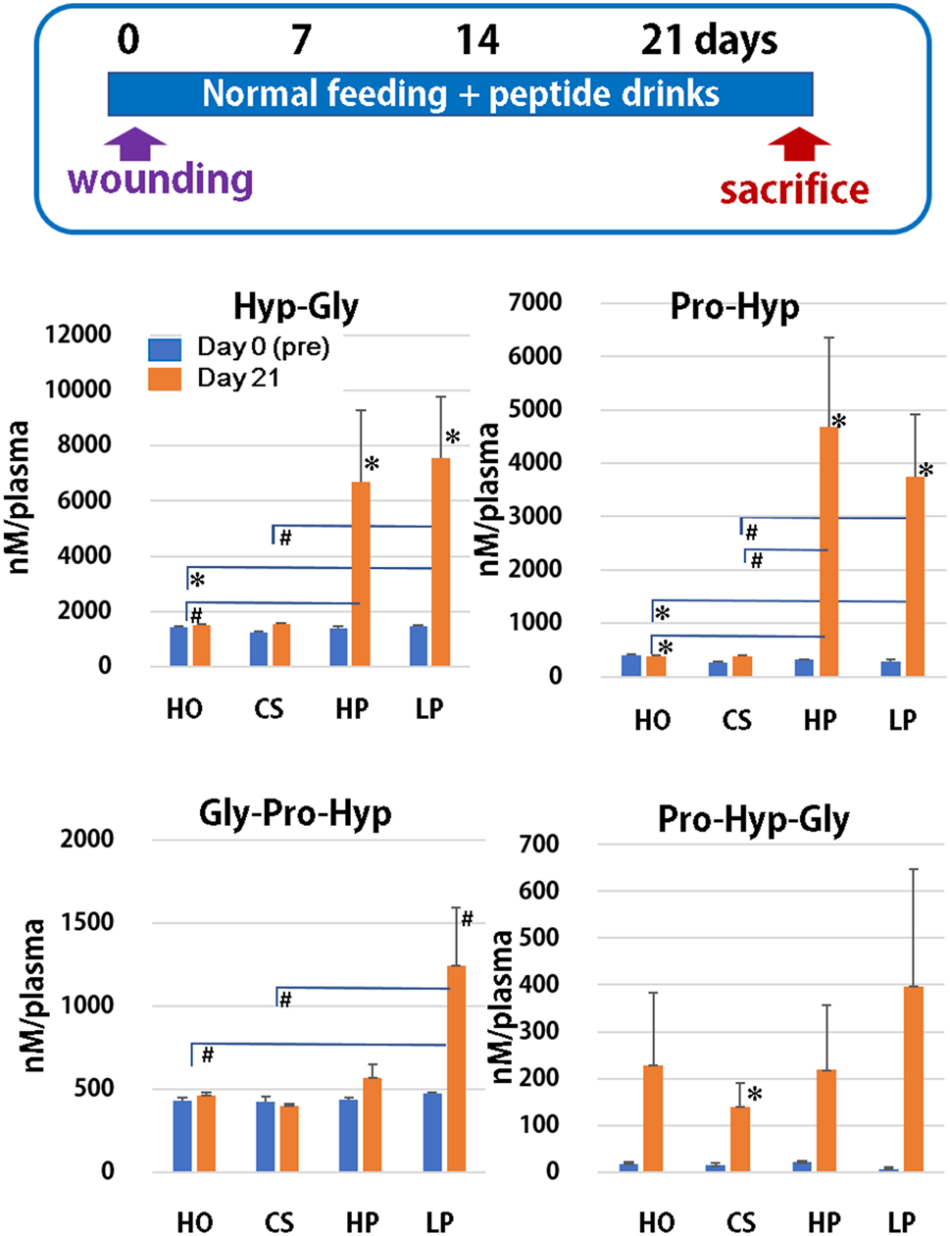
The presence of collagen-derived peptides in the blood after consuming the different peptides. Mice with the abdominal surgery were fed a normal diet and consumed just water (HO) or water that contained milk-derived casein (CS), high molecular weight collagen peptides (HP), and low molecular weight collagen peptides (LP) for 21 days. After the study was completed, collagen peptides in the plasma were measured using a LC–MS/MS technique. Higher contents of peptides were detected in the following groups Hyp-Gly, Pro-Hyp, Gly-Pro-Hyp, and Pro-Hyp-Gly. The comparison was done between the levels on day 0 and day 21 in the same group and between the groups on day 21. N = 5 or 6/group, Mean ± SE, ^#^p > 0.1, *p < 0.05.

Peptide analysis was performed before (day 0) and after the study (day 21) using a LC–MS/MS technique. The major peptides that appeared in the blood were Hyp-Gly, Pro-Hyp, Gly-Pro-Hyp, and Pro-Hyp-Gry, respectively (Fig. 1). Hyp-Gly and Pro-Hyp significantly increased with time in the HP and LP groups, and, on day 21, Pro-Hyp increased in the HP and LP groups compared to other groups. However, no differences in Hyp-Gly and Pro-Hyp were found between the HP and LP groups.

#### Granulation tissue and muscle regeneration

**Figure 2:**
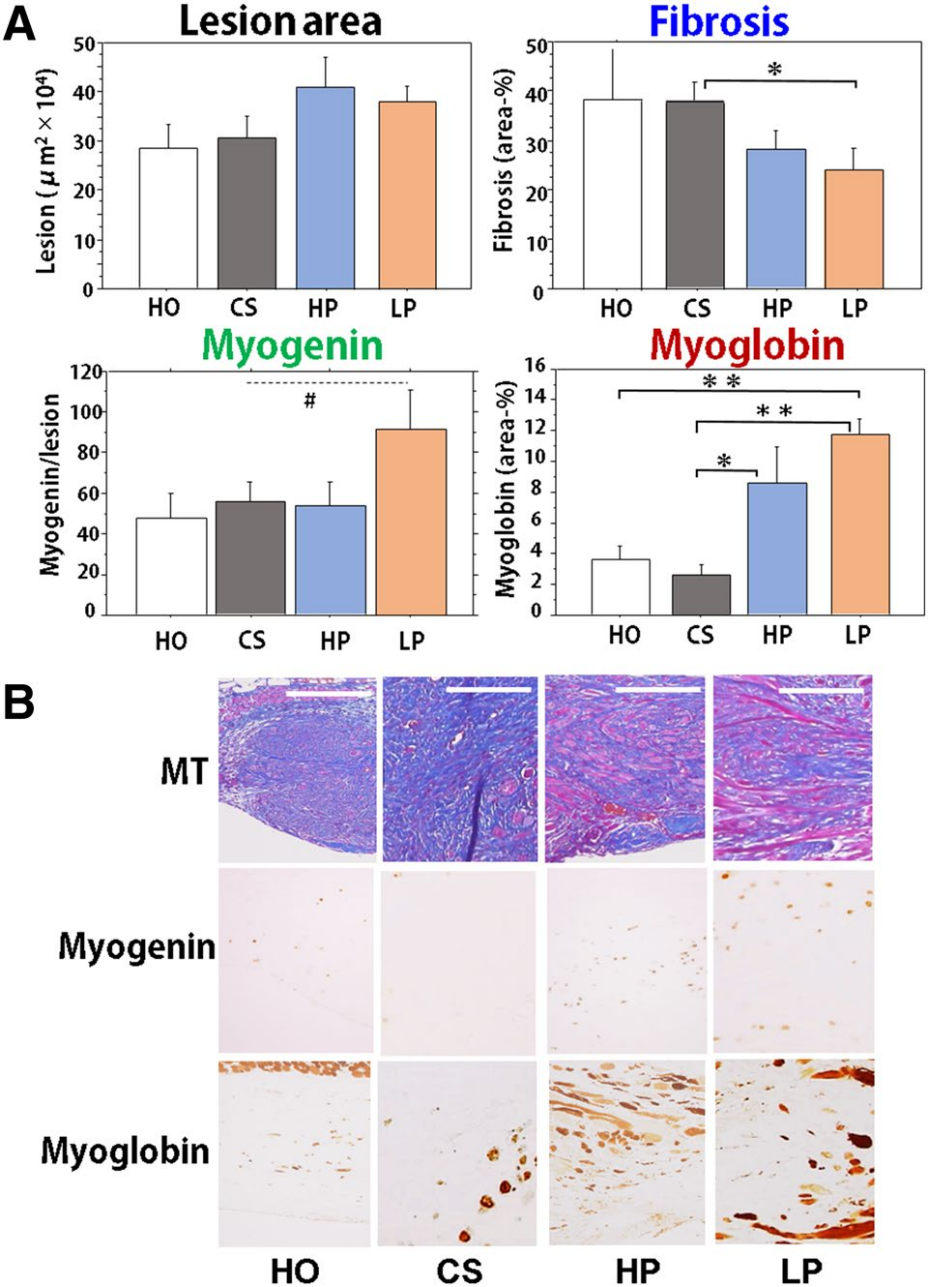
Morphological alterations of granulation tissues after wounding of the abdominal wall in mice that consumed different peptides. (**A**) After the study was completed (day 21), the wound lesion on the abdominal muscle wall was examined for granulation area, fibrosis area, myoglobin positive regenerative muscle area, and myogenin positive cell number in the following groups, HO, CS, HP, and LP. N = 5 or 6/group, Mean ± SE, ^#^p > 0.1, *p < 0.05, **p < 0.01. (**B**) Representative lesions from the HO, CS, LP, and HP groups (day 21). Serial sections were stained by MT stain and immunobiological stains for myogenin and myoglobin. Bars = 100 µm.

On day 21, granulation tissue was developed in the excised lesion between the stumped abdominal muscle wall, and histological analysis was performed. The following parameters were analyzed in the cross-sectional lesion area, fibrosis area using MT stain, myogenin-positive myoblast number, and myoglobin-positive regenerative myocyte area. No differences in the lesion area were found among the groups (Fig. 2A, upper left), however, the fibrosis area was significantly smaller in the LP group than in the CS group (Fig. 2A, upper right; Fig. 2B). In muscle regenerative responses, myoblast number tended to increase in the LP group (p < 0.1 vs. CS) (Fig. 2A, lower left), but regenerative muscle area in the granulation tissue was significantly increased in the LP group (Fig. 2A, lower right), which was followed by the HP group. Representative morphological alterations are shown in Fig. 2B.

### Pro-Hyp intraperitoneal injection experiment

#### Pro-Hyp appearance in the blood after its i.p. injection

Pro-Hyp appeared at its maximum level in the blood 30 min after a single i.p. injection of Pro-Hyp (500 nmol/200 μL) to the mice and then decreased significantly 1 h after the injection (Fig. S2). After 3 h, the level returned to its untreated level.

#### Body weight and hematological alterations

After abdominal muscle wall excision in mice, daily i.p. injection of Pro-Hyp (group PH) or saline control (group SC) was continued for 21 days. No changes were found in body weight, WBC, RBC, and platelet in the blood among the groups (Table S1).

#### Lesion area and fibrosis

**Figure 3:**
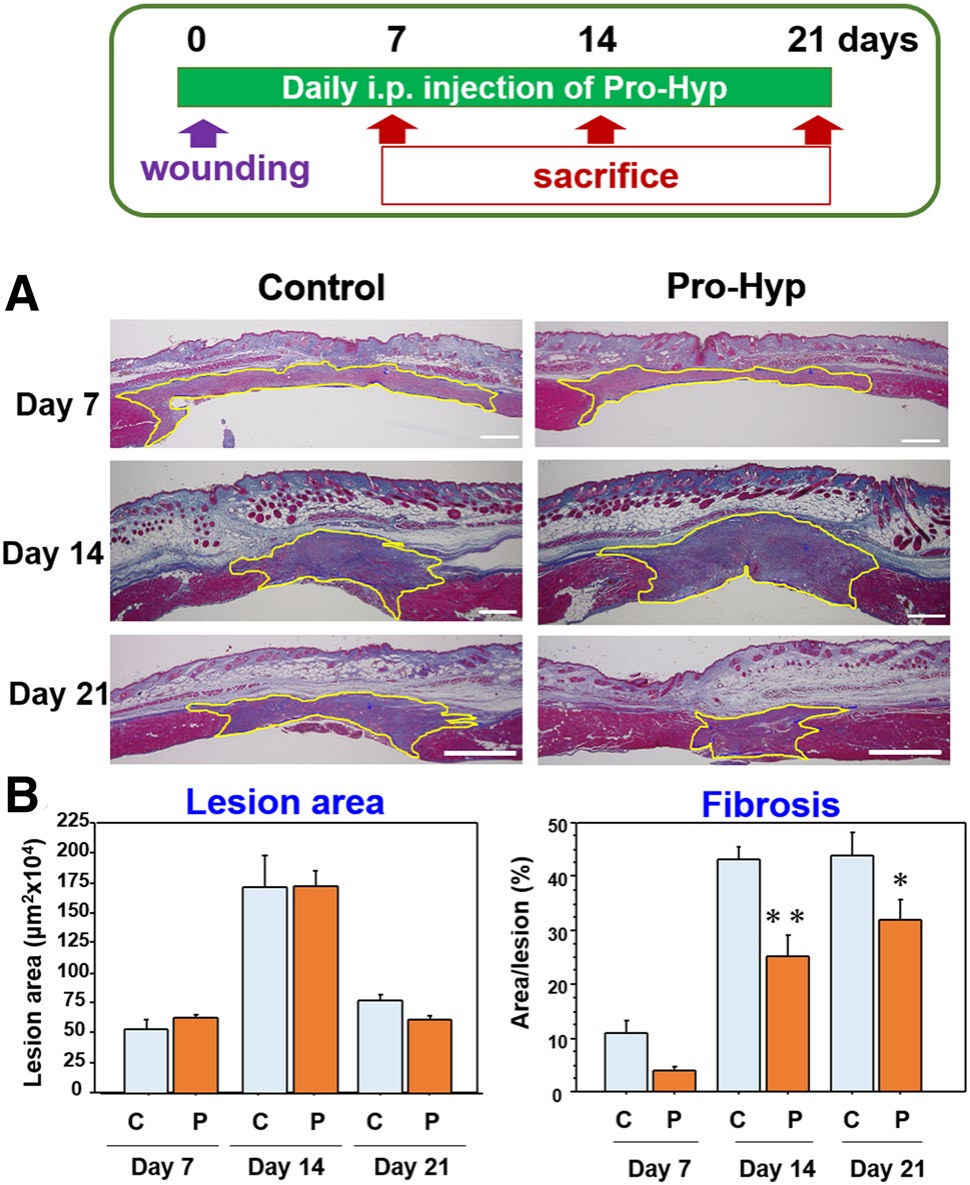
The development of granulation tissue in mice with intraperitoneal Pro-Hyp administration. Pro-Hyp was administered daily i.p. in the mice with abdominal muscle wall excision. The sections of abdominal wound lesion in the saline control group (SC) and Pro-Hyp group (PH) were stained with MT stain (**A**) and examined for granulation tissue area indicated by the yellow line and fibrosis area (%) on days 7, 14, 21 (**B**). N = 5/group, Mean ± SE, *p < 0.05, **p < 0.01. Bars = 500 µm.

During the study, tissue samples were obtained on days 7, 14, and 21. Granulation tissue was developed in the lesion between the stumped abdominal muscle wall under the skin (Fig. 3A), and using MT stain, the total lesion area of the granulation tissue and fibrosis area in cross-section was measured. Lesion areas in both groups similarly changed, but a temporal expansion was seen on day 14, however, no differences were noted between the groups (Fig. 3B, left). On the other hand, fibrosis increased time-dependently in both groups, and in the SC group, it reached the maximum level early on day 14. However, fibrosis level was significantly lower in group PH than group SC after 14 days of the study (Fig. 3B, right).

#### Histological characteristics of muscle regeneration

**Figure 4:**
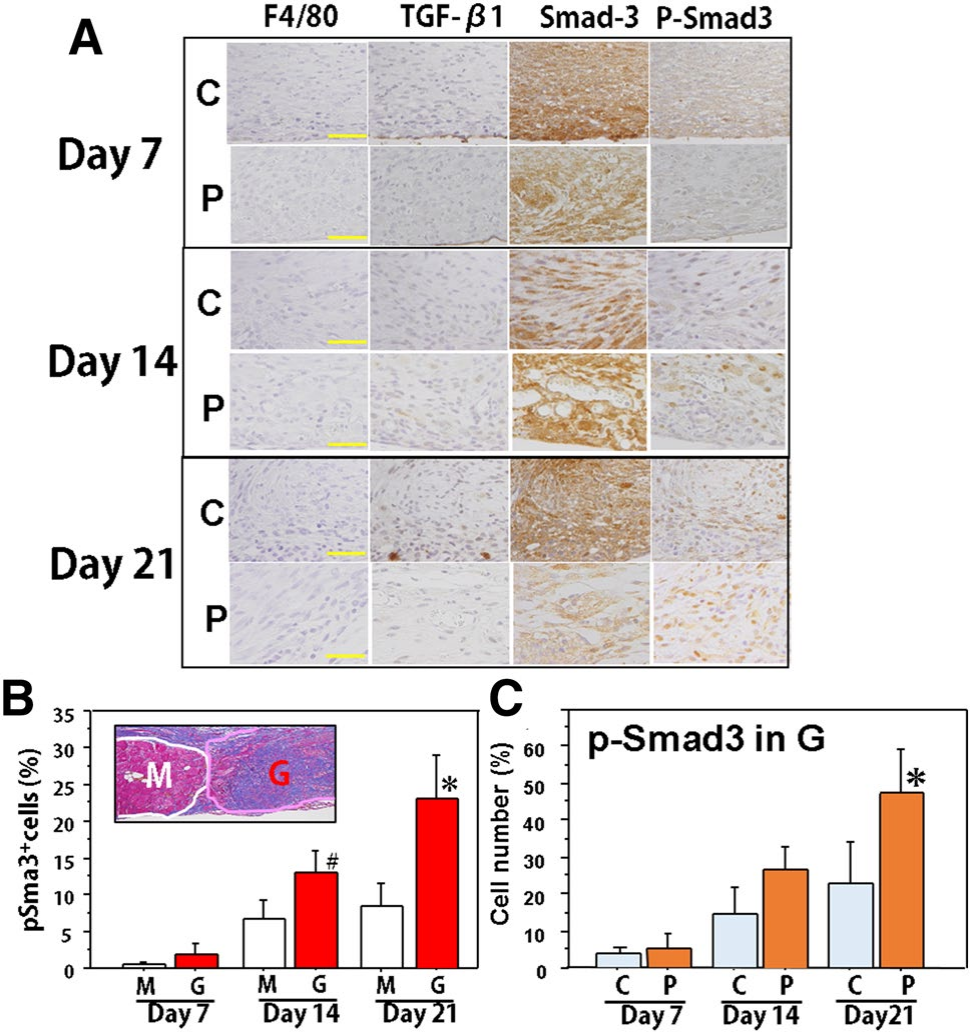
Involvement of the TGF-β-related pathway in the wound of mice in the control and Pro-Hyp groups. (**A**) Representative lesions on days 7, 14, and 21 in the control and Pro-Hyp groups were serially sectioned and stained for F4/80, TGF-β1, Smad3, and p-Smad3. Bars = 40 µm. (**B**) The cells positive for p-Smad3 in the muscle tissue (M) and granulation tissue (G) in the control group were examined on days 7, 14, and 21. N = 5/group, Mean ± SE, ^#^p > 0.1, *p < 0.05. (**C**) The cells positive for p-Smad3 in the granulation tissue were compared between the control and Pro-Hyp groups on days 7, 14, and 21. N = 5/group, Mean ± SE, *p < 0.05.

Muscle regeneration after muscle injury may be initiated by inflammation, growth factors, and cytokines. In this study, TGF-β involving pathways were histologically examined in the granulation tissue on day 21. Inflammatory cell infiltrations including neutrophils (data not shown) and macrophages (F4/80) were scarcely found in the granulation tissue in both groups (Fig. 4A), however, p-Smad3 expression increased over time (Fig. 4A). This phenomenon was detected in both the muscle and granulation tissue (Fig. 4B), with more positive cells in the granulation tissue. Thus, p-Smad3 expression in the granulation tissue was examined. Although all examined cells in the tissue were positive for Smad3 in nuclei, the cells positive for p-Smad3 increased significantly in the Pro-Hyp group compared to the control group on day 21 (Fig. 4C).

#### Fibrosis and muscle regeneration

**Figure 5:**
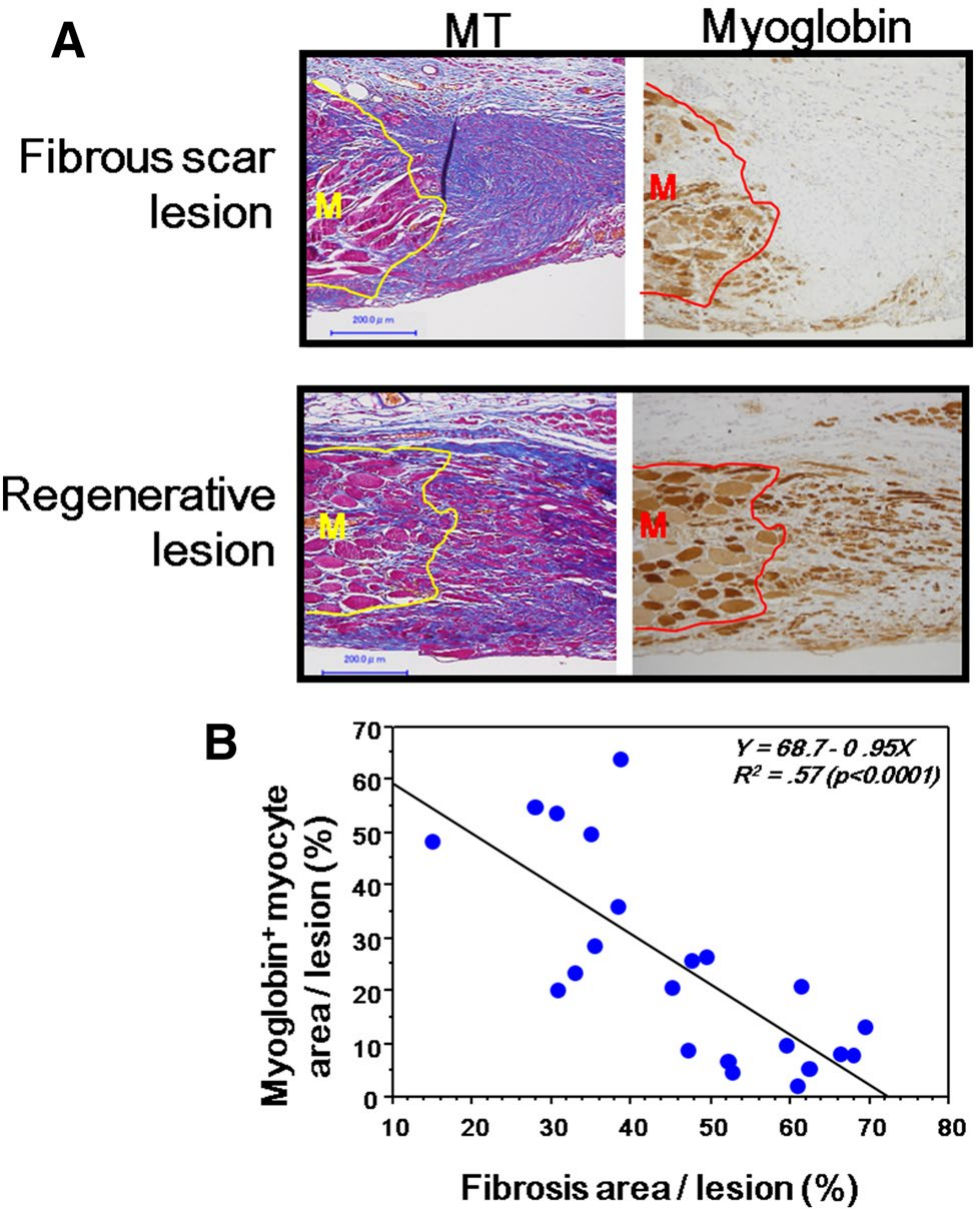
The relation of the fibrous scar lesion and muscle regeneration in wound healing in the abdominal muscle wall. (**A**) The representative lesions of the fibrous scarring and muscle regeneration stained by MT and myoglobin staining using serial sections; The lesion in the upper panel was from the control group on day 21, and the lesion in the lower panel was from the Pro-Hyp group on day 21. The lower panel shows the stumped muscle (M), from which regenerated muscle cells migrate into the granulation tissue. Bars = 200 µm. (**B**) The relation of fibrosis area (%) and myoglobin positive area (%) in the granulation tissue obtained from both groups on day 21.

After abdominal muscle wall excision, granulation tissues were shown to consist of collagen-rich-fibrous elements and muscle regeneration. Among them, representative two different lesions on day 21 from different groups at the muscle-stumped area are shown in Fig. 5A; myogenic regenerative cells migrated from the stumped edge of muscle tissue into the granulation tissue, but when fibrous tissue was formed ahead of the stumped edge, regenerative muscle cell migration was strictly prohibited. We thus analyzed the relationship between fibrosis and myocyte regeneration using mice in both groups on day 21. There is a significant negative correlation between the two parameters (Fig. 5B).

#### Myogenic differentiation in granulation tissues

**Figure 6:**
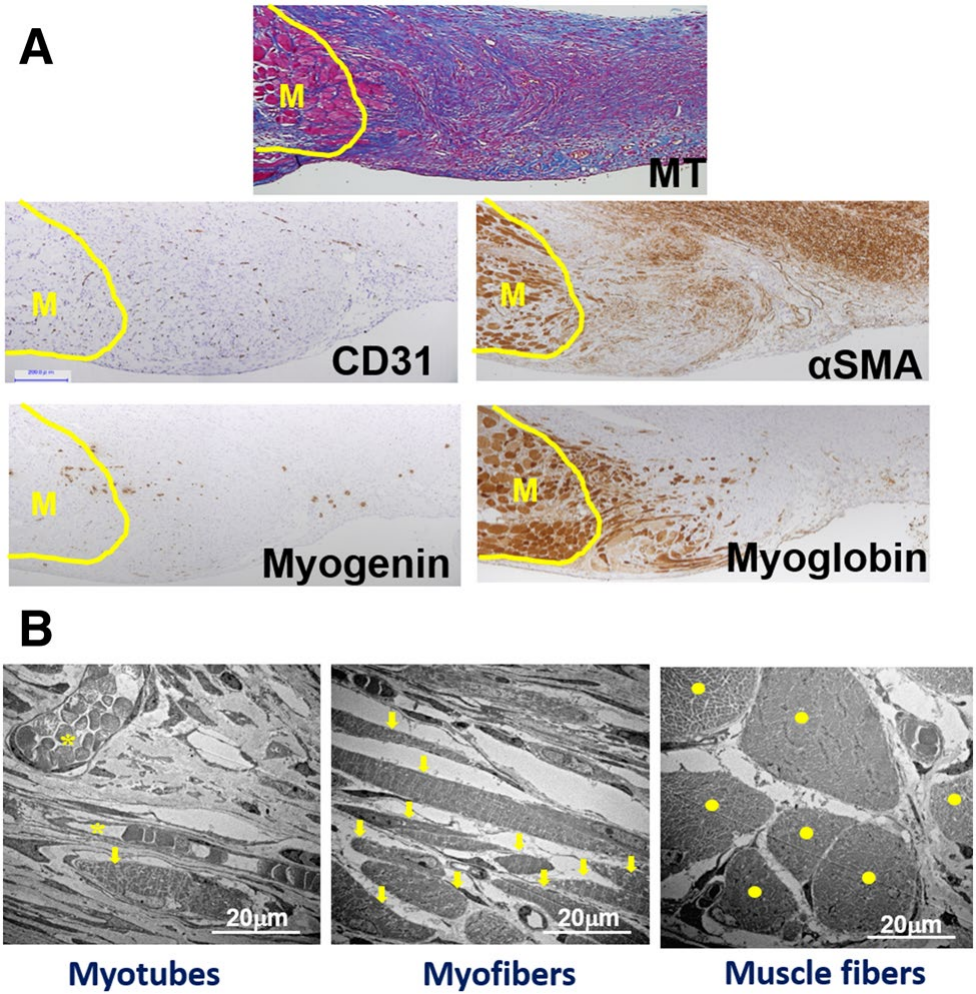
Muscle regeneration from the stumped muscle wall in granulation tissue. (**A**) Distribution of CD31, αSMA, myogenin, and myoglobin positive cells are shown in the granulation tissue with muscle regeneration. The seral sections were obtained from the Pro-Hyp group on day 14. Bars = 200 µm. (**B**) Electron micrographs of the abdominal lesion obtained from the Pro-Hyp group on day 14 showing myogenic differentiation in the granulation tissue. Myotubes forming a multinuclear envelope (left, arrows). Elongated myofibers with transverse striations (meddle, arrows). Large matured muscle fibers (right, dots). Bars = 20 µm.

Figure 6A shows histological alterations in the granulation tissue formed around the stumped muscle tissue on day 14 in the Pro-Hyp group. Fibrosis progressed around the stumped area, but myoglobin-positive muscle regeneration and migration also started from the stumped edge, while myogenin-positive myoblasts were found to scatter near and far from the stumped lesion. αSMA-positive myofibroblasts were densely distributed over the granulation tissue, but not in the muscle-stumped area. CD-31-positive neovessels were densely distributed around the stumped area. To confirm muscle regeneration in the granulation tissue, electron microscopy was performed. Myotubes forming multinuclear cells in an envelope were found (Fig. 6B, left), and elongated myofibers (Fig. 6B, meddle) and large matured muscle fibers (Fig. 6B, right) were also detected in the granulation tissue, but no facia was found.

#### Pro-Hyp administration and muscle regeneration

**Figure 7:**
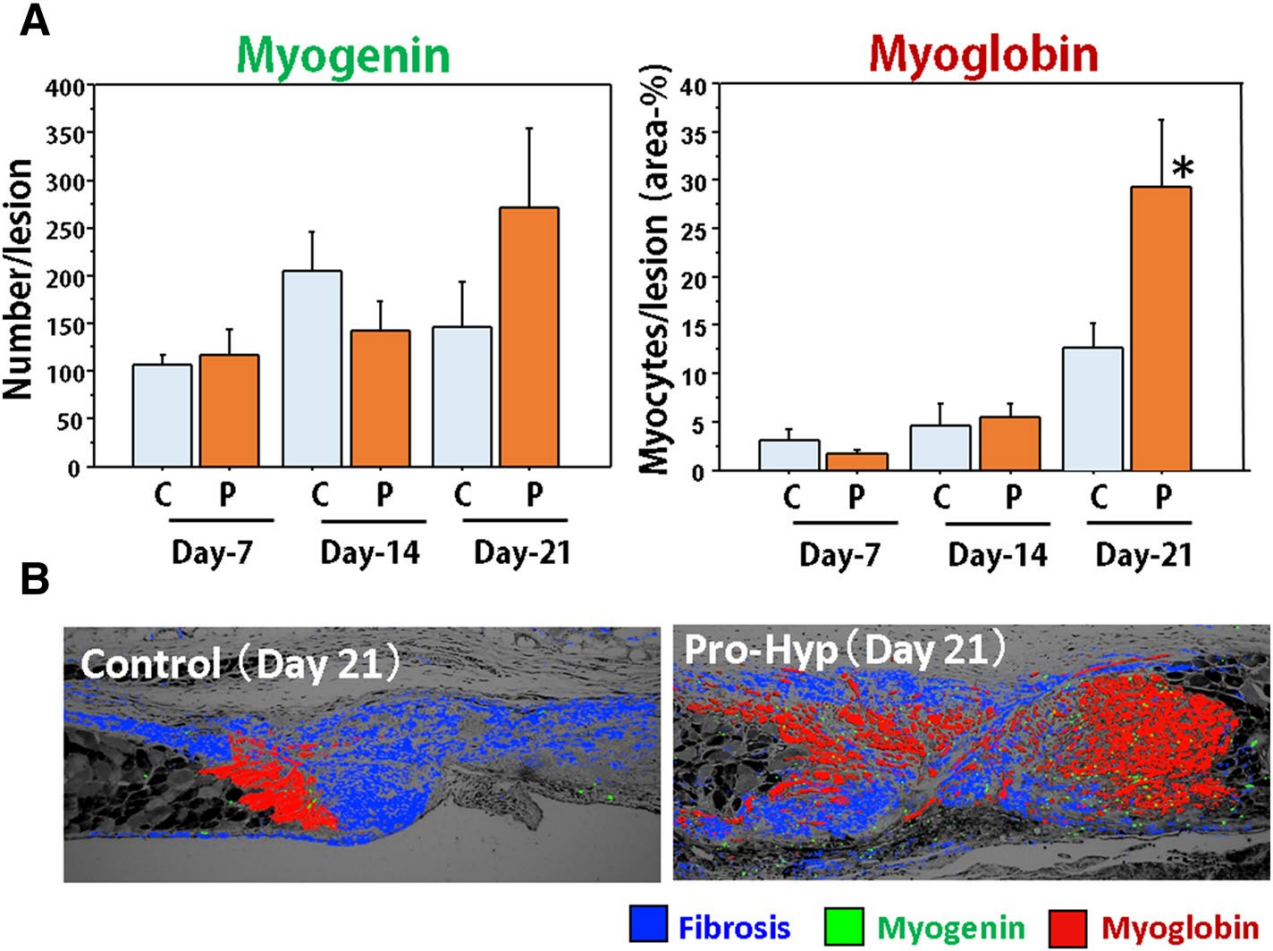
Muscle regeneration in the abdominal wall wounds in mice with i.p. Pro-Hyp administration. (**A**) Myogenin-positive myoblast number and myoglobin-positive regenerative muscle area (%) in the granulation tissue in the control and Pro-Hyp groups on days 7, 14, and 21. N = 5/group, Mean ± SE, *p < 0.05. (**B**) The multiple exposed pictures of granulation tissue on day 21: fibrosis (blue by MT), myoblasts (green by myogenin), and regenerated muscle cells (red by myoglobin). Muscle regeneration is limited to the enormous fibrous lesion in the control group, while regenerated muscle cells spread to all areas of the granulation tissue in the Pro-Hyp group.

The effects of Pro-Hyp on muscle regeneration were analyzed. No statistical differences were found in myoblasts number during the study in groups SC and PH, but the highest level was noted in group PH on day 21 (Fig. 7A, left). Regenerative muscles shown by myoglobin expression significantly increased on day 21 in group PH (Fig. 7A, right). The highlighted images of granulation tissue in the two groups are quite different (Fig. 7B), showing that muscle regeneration is limited in the enormous fibrous lesion detected in group SC, however, regenerated muscle cells spread to all areas of the granulation tissue in group PH. Whereas, no differences in p-Smad3 expression were found between groups PH and SC during the study (data not shown).

## Discussion

It has been known that scar lesions are hardly developed in mice. Our established abdominal muscle-wall excision model, develops fibrosis-prone scar lesions under an in situ tensile force on the abdomen. After 21 days of the drinking study, in granulation tissue, group LP had dominant muscle regeneration with lesser fibrosis among the groups. Although the original level of dipeptides, including Pro-Hyp and Hyp-Gly, in the collagen peptides in the group HP was about 1/30 lower than the group LP, the blood levels of Pro-Hyp after the study showed similar levels in both groups. This discrepancy could be due to Hyp-containing peptides’ resistance against intracellular hydrolysis^24^; it can be therefore speculated that Pro-Hyp and Hyp-Gly were accumulated in the blood even in the mice that consumed HP peptides during the study. Regarding the relocation of administered peptides to the wound lesion, it has been addressed that when Pro-Hyp and Hyp-Gly are orally administrated, Pro-Hyp alone selectively accumulated in the inflamed lesion^25^. Moreover, our previous results showed that Pro-Hyp alone, but not Hyp-Gly, is greatly generated during the development of the granulation tissue^15^, we thus focused on Pro-Hyp, and afterward, daily i.p. injection of pure Pro-Hyp was next performed using the abdominal mouse model. However, it is still possible that low molecule weight collagen peptides other than Pro-Hyp may have a favorable effect on the abdominal wall wound healing.

In the abdominal muscle wall excision model, fibrosis and muscle regeneration simultaneously took place in the granulation tissue. These two pathological changes are contradicted and thus the healing outcome was impacted. In wound healing, granulation tissue formation with collagen matrix is an essential event to reconstitute the defected tissues and provide an emergent scaffold for cell migration and replication. However, if exceeded, lesions would be overwhelmed by collagen-rich fibrous tissues, leading to scar-prone lesions, which may affect subsequent regenerative healings by preventing cellular activities, including neovascularization and stem cell differentiation and proliferation^1,2^. Such morphological transformation in the granulation tissue was evidenced in this study. These alternative changes may affect clinical outcomes in post-surgery patients by forming an unfunctional hypertrophic scars-like collagenous lesion, not only in the skin but also in other organs.

Scar tissues developed in the model may be due to tensile force on the abdominal muscle wall. It has been shown that mechanical stresses alter not only cellular shape and structure but also function^4,5^. It is well established that the pattern of the developed scar lesions in keloid patients is physically followed by the direction of the tensile force on the skin, forming a distinct shape associated with excess fibrosis^2^. Mechanical stresses stimulate and transduce as an intracellular signal, where there are many possible pathways, such as TGF-β/Smad, integrins, MAPK/G-protein, TNF-α/NF-κB, Wnt/β-catenin, interleukin, and calcium-ion channel, by which gene transcriptions for the development of fibrous lesions could progress^26^.

When the skeletal muscle is damaged, various growth factors for muscle repair are involved, including but not limited to, HGF, FGFs, IGFs, PDGF, and TGF-β family^27^. In the process of muscle regeneration after injury, satellite cells in the muscular fasciae start to proliferate and differentiate into myoblasts, and myoblasts fuse with each other, forming myotubes, and they are furthermore maturated into muscle fibers, i.e., myocytes^11,28^. In our study, these cell types were all detected in the granulation tissue that was shown by using morphological and immunohistochemical techniques. This cellular differentiation may be initiated by the factors from not only traumatic tissue damage but also a tensile force on the muscle tissue^29^. Among many possible factors, we focused on the TGF-β/Smad pathway^30^. Although inflammatory cells including macrophages and TGF-β1-possessing cells did not appear in the granulation tissue during the study, p-Smad3 time-dependently increased not only around the stumped muscle tissue but also in the granulation tissue. These results indicate that phosphorylation of Smad3 may occur in the tissues under stimulation of tensile force even in the absence of TGF-β1, and induce progression of fibrosis with collagen deposition, and also activate αSMA expression in myofibroblasts.

A randomized double-blind placebo-controlled study using 120 patients with pressure ulcers indicated that collagen-derived peptides, especially hydrolysates accelerate wound healing reactions^17^. Furthermore, in animal experiments, a diet with Pro-Hyp and Hyp-Gly ameliorate skin barrier defects against skin drying using a mouse model for skin barrier dysfunction^31^. Interestingly, in the skin tissue obtained from the treated mice, up-regulated genes were preferably found in the skeletal muscle-related genes^31^, which could be derived from the cutaneous muscle in the mouse skin, but they unfortunately could not elucidate the phenomenon at that moment. In the present study, Pro-Hyp administration accelerated muscle regeneration after excision of a part of the abdominal muscle wall in mice. These findings strongly support that Pro-Hyp may at a certain point be involved in the myogenic differentiation during the muscle regeneration process.

Intracellular mechanisms of Pro-Hyp effects on cell differentiation are still unclear. However, some mechanisms have been reported; Pro-Hyp induces differentiation in osteoblasts^19^, in which Pro-Hyp binds to Foxg1 and increases Runx2 expression. Runx2 and Smad form a transcriptional complex. In a recent study (2021)^20^, it was shown that Pro-Hyp promotes differentiation of tendon cells with modulation of lineage-specific factors and induces chemotactic activity, proliferation, and type I collagen-network organization. In our recent study using primary fibroblasts derived from mouse skin tissue^32^, Pro-Hyp selectively stimulates migration and proliferation of fibroblasts, which express p75 neurotrophin receptor (p75^NTR^) when cultured on collagen gels. Moreover, nerve growth factor and its receptor p75^NTR^ in myoblasts act for myogenesis and myoprotection when they are damaged^33^. More exact intracellular mechanisms of Pro-Hyp roles especially in myogenic regenerative healing accompanied with reduced fibrosis should be physiologically defined in the future.

## Conclusion

**Figure 8:**
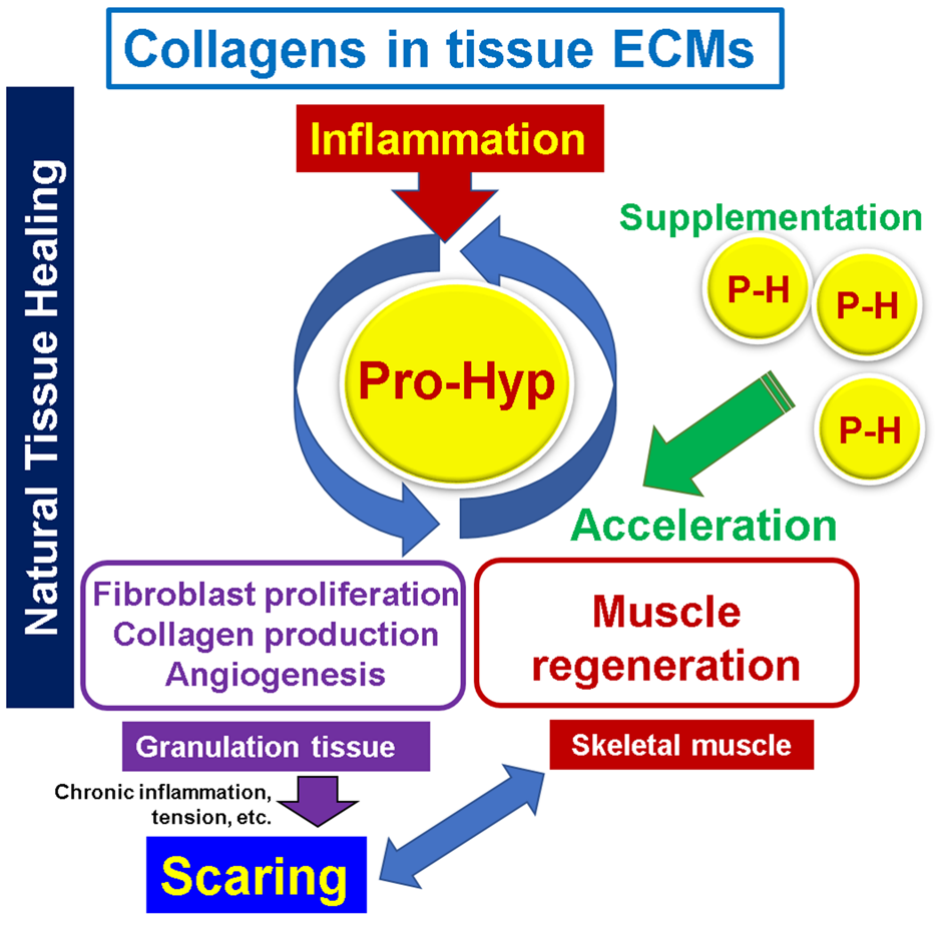
The mechanisms of Pro-Hyp actions on scaring and muscle regeneration in wound healing in the abdominal muscle wall.

The effects of Pro-Hyp were examined using a unique abdominal wound healing mouse model, in which fibrous scarring and myogenic regeneration simultaneously occurred in the granulation tissue formed in the abdominal muscle wall. Pro-Hyp administration, which had no side effects at all, strongly induced migration and differentiation of myogenin-positive myoblasts and myoglobin-positive myocytes in the granulation tissue, but fibrous scar tissue formation was suppressed (Fig. 8). Therefore, it could be effective to administer Pro-Hyp to patients with bedsores, who underwent surgery, or have trauma to activate the proper wound healing process with less scarring. However, additional preclinical studies should be performed.

## Materials and methods

### Materials and chemicals

Collagen peptides manufactured by Nitta Gelatin Inc. Osaka, Japan were used; higher molecular weight collagen peptides (HP): fish collagen peptides (average molecular weight 5000–7000 Da), lower molecular weight collagen peptides (LP): fish collagen peptides (average molecular weight < 1000 Da). Dipeptide content including Pro-Hyp and Hyp-Gly was about 0.1 g/kg in the HP and 3 g/kg in the LP, respectively. Pro-Hyp was purchased from BACHEM (Purity > 98%; Bubendorf, Switzerland). The other analytical grade chemicals were purchased from Sigma-Aldrich Japan Inc. (Tokyo, Japan) and FUJIFILM Wako Chemicals U.S.A. Co. (Osaka, Japan).

### Chemicals for LC–MS/MS

Methanol (high-performance liquid chromatography grade), ammonium acetate (high-performance liquid chromatography grade), and formic acid (high-performance liquid chromatography grade) were purchased from FUJIFILM Wako Chemicals U.S.A. Co. Ammonium bicarbonate was purchased from Sigma-Aldrich Japan Inc. Pro-Hyp and Hyp-Gly were purchased from Bachem (Bubendort, Germany). Standards prepared for Pro-Hyp and Hyp-Gly were dissolved in water, mixed, and diluted to 1 nmol/L, 10 nmol/L, 100 nmol/L, 1,000 nmol/L, and 10,000 nmol/L with 50 mM ammonium bicarbonate buffer. After filtration with a 0.2-mm regenerated cellulose filter (RC4, Sartorius Japan, Tokyo, Japan), 1 mL of the resulting filtrate was used into the LC–MS/MS system.

### Sample preparation for LC–MS/MS

The plasma was deproteinized by adding threefold amounts of 100% (w/v) ethanol. The supernatant was then centrifuged at 13,000 rpm for 10 min at 4 °C. The supernatant was diluted tenfold with 50 mM ammonium bicarbonate buffer. After filtering through a 0.2-mm regenerated cellulose filter, the resulting filtrate was injected into the LC–MS/MS system.

### LC–MS/MS analysis

Samples were analyzed by LC–MS/MS. The LC analysis was performed using an ACQITY UPLC H-class Biosystem (Waters, Milford, MA, USA). The MS/MS analysis was performed using a Xevo TQ-XS system (Waters, Milford, MA, USA). Hypersil GOLD PFP column (2.1 × 150 mm, 5 μm, Thermo Fisher Scientific Inc., Tokyo, Japan) was used for separation. Gradient elution was carried out: A buffer with 0.2% (v/v) formic acid and 2 mM ammonium acetate and B buffer with 100% methanol. The gradient profile with the following proportions (v/v) of B buffer was applied (t (min), % B buffer, flow rate): (0 min, 2%, 200 µL/min), (3.50 min, 2%, 200 µL/min), (3.51 min, 95%, 400 µL/min), (7.00 min, 95%, 400 µL/min), (7.10 min, 2%, 200 µL/min) (10 min: time was required to reach initial conditions). The column temperature was maintained at 40 °C. The tandem quadrupole mass spectrometer was used in positive ion electrospray mode. The ion source was operated at 150 °C with a capillary voltage of 1.5 kV. Nitrogen was employed for the desolvation gas at 500 °C. The mode of acquisition was multiple reaction monitoring (MRM) at an argon collision gas pressure of 3.5–3.8 × 10^–3^ mbar. The list of peptides and the MRM transitions and collision energies for the method are presented below. The data were acquired using MassLynx Software version 4.2 (Waters, Milford, MA, USA).

**Table:** 

	MRM (m/z, precursor > product)	Collision energies
Pro-Hyp	229 > 132	15
Hyp-Gly	189 > 86	15

### Animals

Animal experiments were conducted with the approval of the Fukuoka University Animal Experiment Committee (No. 1812097) on December 18, 2018, and study protocols complied with the institution’s animal care guidelines. Eight to 10-week-old C57BL/6 female mice (Japan SLC Inc., Shizuoka, Japan) were used. All procedures were conducted under aseptic conditions, using autoclaves, ethylene oxide gas, 70% ethanol, and povidone-iodine. Mice were anesthetized with pentobarbital (Somnopentyl; KYORITU SEIYAKU, Tokyo, Japan). Hematological analyses, including red blood cell (RBC), and white blood cell (WBC) counts (Celltac-α, NIHON KOHDEN, Tokyo, Japan) were done in the blood drawn from the eyelids using a heparinized 75-µL capillary (Hirschmann Laborgeräte GmbH & CO., Eberstadt, Germany).

### Abdominal muscle wall excision model

The model was established; In brief, after removing hair on the abdomen, mice under general anesthesia were set down in a supine position, and a 1.5 cm line alongside the midline in the abdominal skin was marked. After applying iodine to the abdominal area, the skin was resected along the marked line. After separating the skin around the cut line from the abdominal muscle wall with scissors, the fascia at the umbilicus spot was pulled up and a needle with a suture was inserted. The tissue was then lifted with the thread and the spot, where the suture had been inserted, was grasped with round-top tweezers. The tissue was cut away around the circular edge of the tweezers, and the amounts of excised tissue were all set in more than 6 mg. The opened skin was placed together and sutured at upper and lower positions. The abdominal area of the wound was covered with a film dressing (Tegaderm; SUMITOMO 3 M, Tokyo, Japan). After the surgery, a silicon vest was placed on the mice, and the vests were kept on the mice for 1 week to complete surficial skin healing. After 1 week, the vest was removed.

### Pro-Hyp administration

#### Drinking experiment

Different molecular weight collagen peptides HP and LP, and milk-derived Casein (CS) (Sigma-Aldrich Japan, Tokyo, Japan) were dissolved at a concentration of 8% in water and autoclaved. The solutions and water alone (HO) were given to mice without restriction. Each group had 5 or 6 mice and 2 or 3 mice were housed in the same cage with a normal diet. The amount of solution and body weight were monitored every other day during the 21 days of the study. On day 21, blood was obtained for the quantitative analysis of peptides using the LC–MS/MS method. Thereafter, the mice were sacrificed, and the abdominal tissue was harvested and photographed with a digital camera (NEX-C3; SONY Co., Tokyo, Japan) and used for histological examination.

#### Intrapreneurial administration experiment

Intraperitoneal injections of 500 nmol per 200 µL Pro-Hyp (group PH) or 200 µL saline (group SC) were administered daily. On days 7, 14, and 21, blood was obtained for the general hematological analysis, and the blood on day 21 was used for the quantitative analysis of peptides using the LC–MS/MS method. Thereafter, the mice were sacrificed, and the abdominal tissue was harvested and photographed with a digital camera (NEX-C3; SONY Co., Tokyo, Japan) and used for histological examination. Seven mice were used in each group in different time points. In some analyses, the plasma level of Pro-Hyp after a single i.p. injection of Pro-Hyp was monitored for 7 days (n = 3). Saline injection was used as a control (n = 3).

### Histological examination

The tissue was fixed in 5% buffered formaldehyde (pH7.4) for several days. Two pathological tissue samples from the wound were excised and embedded in paraffin blocks using a tissue processor (ASP200S; LEICA Biosystems, Nussloch, Germany). Four-µm-thick tissue sections were cut with a microtome (LEICA RM2235; LEICA Biosystems).

### Histological staining

Tissue sections were stained with hematoxylin and eosin (HE) and Masson’s trichrome (MT) stains. For immunohistological examination, the following primary antibodies were used: rat anti-NIMP-R14, a Ly-6G/-6C neutrophil marker (1:400; Hycult Biotech, Netherlands), rat anti-CD31 (1:30; Dianova GmbH, Hamburg, Germany), rat anti-F4/80 (1:200; Abcam plc), rabbit anti-TGF-β1 (1:800; Abcam plc) rabbit anti-αSMA (1:2000; Abcam plc), rabbit anti-Smad3 (1:1000; Gene Tex Inc., CA, USA), rabbit anti-phosphorylated Smad3 (p-Smad3) (1:8000; Rockland Immunochemicals Inc., Limeric, PA), rabbit anti-myoglobin (1:1000; Abcam plc), and rabbit anti-myogenin (1:200; Abcam plc). All were diluted with Antibody Diluent (DAKO Japan Inc., Tokyo, Japan). For the CD31, TGF-β1, myoglobin, and myogenin antibodies, antigens were activated by heating in 0.01 M citrate buffer (pH 6.0) for 5 min. Sections were incubated overnight with the primary antibody at 4 °C. The immunobound antibodies were detected using biotin-conjugated secondary antibodies in the N-Histofine Simple Stain Mouse MAX PO (Rat) detection system (Nichirei Biosciences Inc., Tokyo, Japan) and the EnVision kit (DAKO Japan Inc.), and the sections were developed by 3,3′-diaminobenzidine stain and counterstained with hematoxylin. Stained sections were observed under a microscope (Bio-Zero and BZ-X800; KEYENCE Co.).

### Morphometrical analysis for granulation tissue

Total lesion area: Using histological pictures stained by HE and MT stains, the area of granulation tissue developed between the abdominal muscle wall, including fibrosis and regenerative and migrated muscle cells, was morphometrically measured with a computer-assisted-software BZ-II (Keyence).

Fibrosis area: representative lesions in MT stain were taken using 10-time lens in the Bio-Zero (Keyence), and the blue staining collagenous fibers were highlighted, binarized, and measured (VH-analyzer: Keyence, Osaka, Japan), and the percentage of collagen distribution in the granulation tissue was calculated.

Myoblast number: Myogenin-stained granulation tissue was taken using a 10-time lens, and the positive spots in the area of granulation tissue were calculated.

Regenerated myocyte area: Myoglobin-stained granulation tissue was taken using a 10-time lens, and the positive area of regenerative muscles in the granulation area was calculated.

Positive cell number for p-Smad3: Cells with a positive nucleus for p-Smad3 were counted out of the cells in tissue photographed with a 20-time lens. Examined tissues were of the granulation tissue and the stumped muscle (Fig. 7B).

### Statistical analysis

Numerical results are presented as the mean ± standard error (SE) or standard deviation (SD). Two groups were compared using a Student’s t-test, and a linear regression analysis was performed to compare the two values. P values less than 0.05 were considered significant. P values less than 0.05 and 0.01 are indicated by the asterisk (*) and (**) in the graphs, respectively.

## Supplementary Information

Supplementary Information.

## Data Availability

This study is reported in accordance with ARRIVE guidelines (https://arriveguidelines.org). Patents: JP 6709440: Hypertrophic Scar Inhibiting Compositions.
