# Supplementary material for: Collagen-derived dipeptide Pro-Hyp administration accelerates muscle regenerative healing accompanied by less scarring after wounding on the abdominal wall in mice
Source: Sci Rep. 2021 Sep 21;11:18750. doi: 10.1038/s41598-021-98407-9 (PMC8455591; doi:10.1038/s41598-021-98407-9)
Supplement: Supplementary file 1 — Supplementary Information. [file 41598_2021_98407_MOESM1_ESM.pdf]

Fig. S1. Drinking amounts per mouse with different peptide drinks.

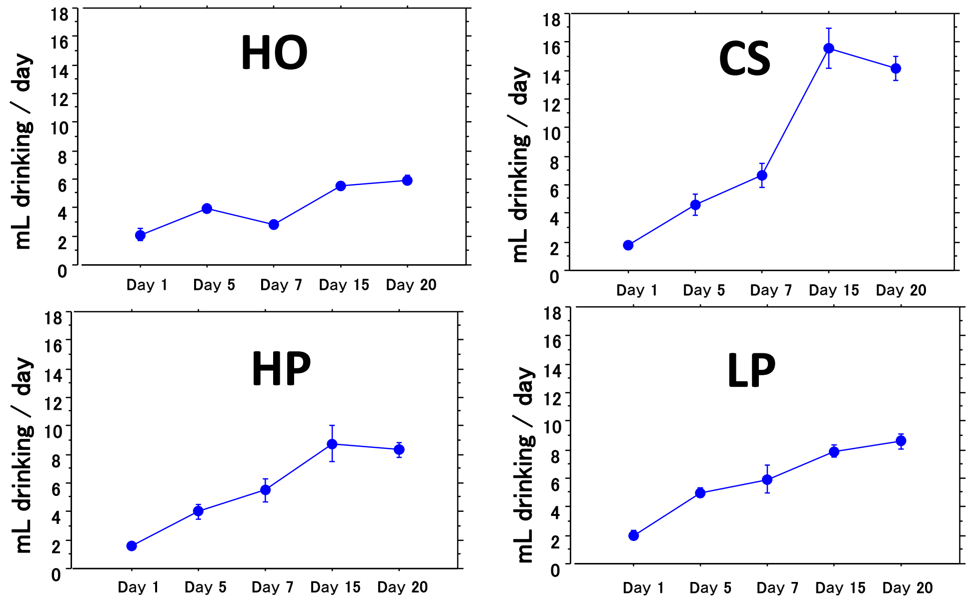

Table S1. Bodyweight and blood profiles in abdominal wound model mice with different peptide drinks.

		Day 0	Day 8	Day 15	Day 21
Bodyweight g	HO	18.6±1.0	18.2±1.3	20.5±1.3	21.2±1.2
	CS	18.8±0.7	17.6±0.9	19.7±0.7	20.9±0.5
	HP	18.5±0.7	18.2±0.6	20.3±0.6	21.1±0.8
	LP	18.9±0.7	18.1±0.9	20.1±0.9	20.8±0.5

		Day 0	Day 8	Day 15	Day 21
WBC $\times 10^2/\mu L$	HO	41.2±9.2	56.3±11.7	29±18	49±13.1
	CS	36.6±10.3	48±19.5	37.2±1.8	40±13.1
	HP	44.7±10.5	55.5±10.5	34±7.7	51±14.2
	LP	39.5±15.5	48.214.3	29±18	46.5±14.9

		Day 0	Day 8	Day 15	Day 21
RBC $\times 10^4/\mu L$	HO	1118±36	900±74	971±54	1091±94
	CS	1160±50	886±64	1043±69	1067±103
	HP	1141±21	923±93	1010±37	1070±80
	LP	1148±37	929±46	1015±25	1003±42

		Day 0	Day 8	Day 15	Day 21
PLT $\times 10^4/\mu L$	HO	73.3±9.6	51.4±10.2	98.6±18.3	88.2±8.4
	CS	81±7.8	53.3±17.5	86±25.7	75.6±18.2
	HP	74.8±10.8	66.3±22.9	90±16.3	78.2±14
	LP	76.4±6.7	61.6±13.4	80.8±19.3	79±15.1

(Mean ± SD)

Fig. S2. Plasma Pro–Hyp concentrations after a single i.p. injection of Pro–Hyp.

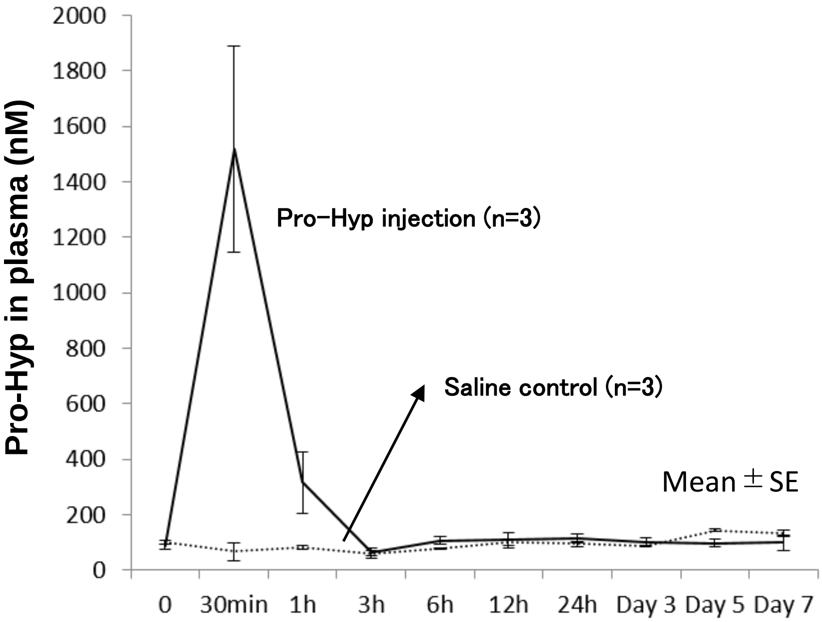

Table S2. Bodyweight and blood profiles in abdominal wound model mice with daily i.p. injection of Pro–Hyp.

		Day 0	Day 7	Day 14	Day 21
Bodyweight g	Control (C)	19.5 ± 0.7	17.9 ± 1.0	17.7 ± 2.3	21.1 ± 1.2
	Pro–Hyp (P)	19.5 ± 0.8	17.5 ± 0.4	17.8 ± 1.0	21..0 ± 0.8
		Day 0	Day 7	Day 14	Day 21
WBC $\times 10^2/\mu L$	Control (C)	74 ± 22	53 ± 7	63 ± 12	82 ± 14
	Pro–Hyp (P)	48 ± 7	40 ± 13	48 ± 6	74 ± 12
		Day 0	Day 7	Day 14	Day 21
RBC $\times 10^4/\mu L$	Control (C)	950 ± 76	1016 ± 34	1006 ± 34	926 ± 74
	Pro–Hyp (P)	984 ± 50	941 ± 93	939 ± 55	873 ± 44
		Day 0	Day 7	Day 14	Day 21
PLT $\times 10^4/\mu L$	Control (C)	65 ± 11	76 ± 8	70 ± 13	50 ± 22
	Pro–Hyp (P)	50 ± 21	90 ± 8	82 ± 6	65 ± 9

(Mean ± SD)
